# Ups and downs in catch-up saccades following single-pulse TMS-methodological considerations

**DOI:** 10.1371/journal.pone.0205208

**Published:** 2018-10-11

**Authors:** James Mathew, Frederic R. Danion

**Affiliations:** Aix Marseille University, CNRS, Institut de Neurosciences de la Timone UMR 7289, Marseille, France; Universita degli Studi di Verona, ITALY

## Abstract

Transcranial magnetic stimulation (TMS) can interfere with smooth pursuit or with saccades initiated from a fixed position toward a fixed target, but little is known about the effect of TMS on catch-up saccade made to assist smooth pursuit. Here we explored the effect of TMS on catch-up saccades by means of a situation in which the moving target was driven by an external agent, or moved by the participants’ hand, a condition known to decrease the occurrence of catch-up saccade. Two sites of stimulation were tested, the vertex and M1 hand area. Compared to conditions with no TMS, we found a consistent modulation of saccadic activity after TMS such that it decreased at 40-100ms, strongly resumed at 100-160ms, and then decreased at 200-300ms. Despite this modulatory effect, the accuracy of catch-up saccade was maintained, and the mean saccadic activity over the 0-300ms period remained unchanged. Those findings are discussed in the context of studies showing that single-pulse TMS can induce widespread effects on neural oscillations as well as perturbations in the latency of saccades during reaction time protocols. At a more general level, despite challenges and interpretational limitations making uncertain the origin of this modulatory effect, our study provides direct evidence that TMS over presumably non-oculomotor regions interferes with the initiation of catch-up saccades, and thus offers methodological considerations for future studies that wish to investigate the underlying neural circuitry of catch-up saccades using TMS.

## Introduction

Transcranial magnetic stimulation (TMS) has been proven a useful technique to investigate noninvasively the neural circuitry underlying eye movements, such as visually guided saccades, and smooth pursuit [1,2]. In reaction time protocols, depending on the timing of TMS with respect to the target appearance and/or expected saccade onset, TMS can sometimes shorten or increase the reactive saccade latency [3,4]. In this context, TMS over brain areas such as the frontal eye field (FEF) [5–7], or the cerebellum [8,9] has been shown particularly effective. However some studies report that TMS over the vertex can also be effective [3,4], thereby suggesting that some of these effects are not necessarily restricted to a single brain area, and can extend beyond well-established oculomotor regions. Regarding smooth pursuit activity, TMS has been shown to induce increase or decrease in pursuit velocity depending on target dynamics, when being applied over brain regions such as FEF [10,11] or the cerebellum [12,13].

Despite the fact that many studies have been performed to document the effect of TMS on smooth pursuit and visually guided saccades, to our knowledge, there are no studies that investigated the effect of TMS on catch-up saccades, a special type of saccade initiated during smooth pursuit when position and/or velocity error become too important [14]. The goal of the current study is to provide preliminary information regarding the possible effect(s) of TMS on catch-up saccades. To achieve this goal we reanalyzed the data collected in our recent study [15] that focused on the effect of TMS over the primary motor cortex (M1) during smooth pursuit activity. In that study our protocol included two main situations, one in which the eyes had to track a visual target that followed a trajectory determined by an external agent, and one in which the target was moved by the participants’ hand, a condition known to decrease the occurrence of catch-up saccade [16–18]. As will be shown later, because we have found that TMS over M1 had an impact on the initiation of catch-up saccade, we have decided to analyze additional data from the same participants in which TMS was applied over the vertex, a brain region presumably even less concerned with eye movements than M1. The current study will demonstrate that, following M1 or vertex stimulation, there is a consistent modulation of catch-up saccade activity no matter whether participants track an externally or a self-moved target. Overall the robustness of our findings emphasizes the necessity to include control sites in future studies that wish to investigate the neural circuitry of catch-up saccades with TMS.

## Methods

As a more detailed description of the equipment, experimental design and tasks can be found in our previous study [15], here we only report crucial information. Note that TMS could be triggered in two different ways in our previous study: either by the target kinematics (main experiment) of by the eye kinematics (control experiment). Here we focus on the data collected during the control experiment, but similar modulatory effects of TMS of saccadic activity are obtained with the data from the main experiment. Please also note that we present new data from the same participants investigating the effect of TMS over the vertex (not shown in our earlier study).

### Participants

Six healthy right-handed volunteers were recruited (age: 27.3 ± 10.0, hereinafter mean ± standard deviation 5 male). Half of them participated to the main TMS experiment [15] which was performed one year prior to this control experiment. These 3 participants were chosen randomly from the original pool of subjects. Several days before the experiment, participants received written and oral information about the TMS technique, and underwent examination to confirm that they had no contraindications to TMS [19]. All participants gave written consent prior to participation and received 40€. The experimental paradigm (N°2013–1346) was approved by local ethics committee called "Comité de Protection des Personnes Sud Méditerranée 1", and complied with the Declaration of Helsinki.

### Setup

**Fig 1:**
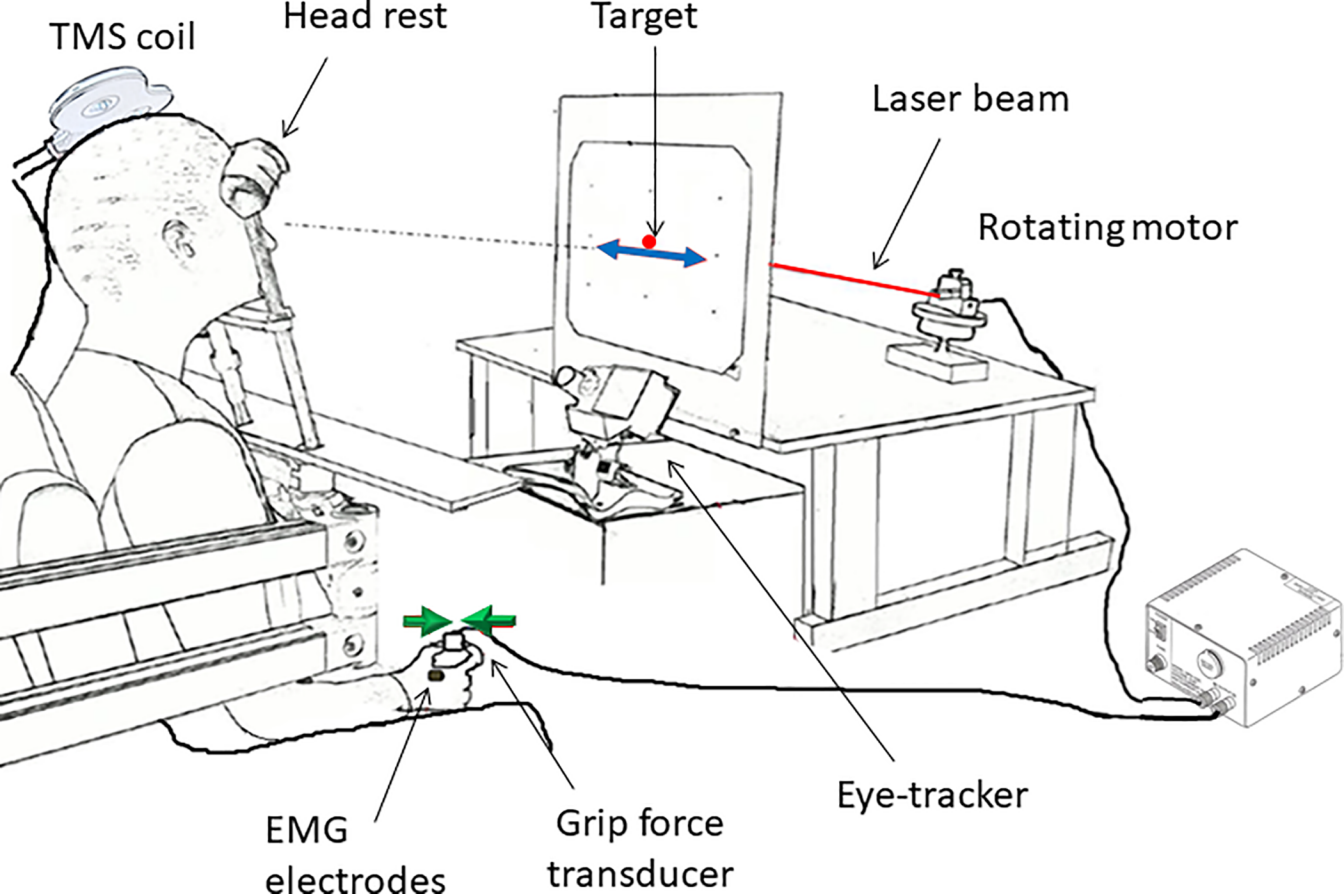
Schematic drawing of the experimental setup. All parts of this figure were drawn by the authors. This figure is similar but not identical to the original image in [15] and is therefore for illustrative purposes only. See text for more details.

A drawing of the experimental setup is shown in Fig 1. Being comfortably seated in a dark room, participants faced a screen positioned on the frontal plane 57 cm away from the eyes. A mask positioned under the participants' chin blocked vision of the hands. In some of the experimental conditions participants were required to grasp with the right hand a force sensor (ELPM-T1M-25N, Entran, Fairfield, NJ) between the index finger and the thumb. Electromyographic activity was recorded from the first dorsal interosseous. The target was a red disk (0.5° in diameter) projected on the screen by means of a laser beam moved by a servo-controlled optical scanner (delay < 2 ms). Right eye movements were recorded using an infrared video-based eye tracker (Eyelink Desktop-mounted system, SR Research). All signals were collected at 1000 Hz.

### TMS hotspot search

A figure of eight double coil (70 mm) connected to a Magstim Bistim2 magnetic stimulator (Magstim, Whitland, UK) was positioned tangentially to the scalp, and oriented perpendicular to the central sulcus and 45° angle to the interhemispheric fissure [20,21]. The site at which the largest grip force pulse elicited was determined while the participant held a constant grip force (GF) of 3N. When being located we determined the corresponding active motor threshold (AMT) at that grip force level. Mean group AMT was 40.0 ± 4.9% of maximum stimulator output. During the experiment TMS intensity was set at 52.8 ± 6.7% of maximum stimulator output (130% of AMT), resulting in grip force pulses of about 2N when holding a constant GF of 3N. When investigating the stimulation of the vertex, the coil was placed over the central midline (or Cz according to the EEG 10–20 electrode system). As expected, TMS over the vertex did not induce any modulation of grip force.

### Experimental design

In all trials, participants were asked to track with their eyes a moving target on the screen as precisely as possible. Depending on the experimental condition, target motion was driven either by the participants’ hand (SELF) or by an external agent (EXTERNAL). When participants drove the target motion through grip force modulations (SELF protocol), the mapping was chosen such that, when GF = 3N the target was positioned at the center of the screen, when GF = 5N the target moved 15° to the right (+), and when GF = 1N the target moved 15° to the left (‒). Participants were instructed to perform random oscillatory target movements (for a similar procedure see [17,18,22]). The rational was to make target motion as unpredictable as possible when being subsequently played back in the EXTERNAL conditions (see later).

A total of 3 conditions were tested with a self-moved target. In the first one called SELF, the participants had to move the target randomly with the hand and track it with the eyes. In the SELF-M1 condition, the task was identical but intermittently (4 times per trial) TMS pulses were triggered (see more details later). Despite that the effect of TMS on grip force induced fast target jumps, participants were encouraged to keep on tracking the target. In the SELF-VERTEX condition, the task was identical except that this time TMS was triggered over the vertex, and therefore did not induce target jumps.

Regarding trials under which target motion was externally driven (EXTERNAL), a total of 3 conditions were tested. In the first one, called EXT, target trajectories collected during SELF were played back. Each participant was presented his/her own trials. In the second condition, called EXT-M1, a similar procedure was used except that occasionally (4 times per trial) TMS was triggered over M1. In the third one, called EXT-VERTEX, the procedure was similar to EXT-M1 but this time TMS applied over the vertex. Overall we explored a total of 6 experimental conditions. Each participant performed 1 block of 15 trials (30s each) in each of these experimental conditions. The order of the blocks was randomized except the SELF block which had to be completed first before participants could perform the EXT, EXT-M1, and EXT-VERTEX blocks. To comply with this randomization of the blocks, the coil was repositioned over M1 or the vertex within a few minutes.

### Timeline of TMS

TMS was triggered by current eye velocity which had to be in the vicinity of 20°/s (range: 17–23°/s) for at least 10 ms. This method was intended to ensure that TMS was triggered in comparable states of the eye, but also to prevent that TMS was triggered during an ongoing catch-up saccade. TMS was applied four times within each trial: two during a rightward target motion, and two during a leftward target motion, with an order that was randomized across trials. We also imposed that TMS could not be triggered during the first 4 seconds, and that consecutive TMS pulses were separated by at least 4.5 seconds.

### Data analysis

We first performed a sequence of analyses to separate periods of smooth pursuit, saccades and blinks. The identification of blinks led to the removal of about 1% of eye recordings. Eye position time series were then low-pass filtered with a Butterworth (4th order) using a cutoff frequency of 25 Hz. The resultant eye position signals were differentiated and were low-pass filtered with a cutoff frequency of 25 Hz to remove the noise from the numerical differentiation. The resultant eye velocity signals were also differentiated and low-pass filtered at 25 Hz to provide acceleration traces. A dedicated Matlab script identified the beginning and end of each saccade based on acceleration and deceleration peaks (>1500°/s^2^). Subsequent visual inspection allowed to detect saccades (<1°) that could not be identified by our program.

To assess the effect of TMS on the initiation of catch-up saccades the probability to observe a saccade (or fraction of a saccade) was computed for each of the 20 ms time bin that composed the time interval starting 40 ms before TMS and finishing 340 ms after TMS. For comparison purposes, saccade probability was also computed for equivalent time periods when there was no TMS (but in which TMS could have been triggered given our criteria on eye kinematics). The accuracy of catch-up saccades was also examined in the same time interval by measuring the absolute distance between eye and target position at the end of saccade. Because the effect of TMS on catch-up saccades was similar when it was triggered during rightward or leftward target motion, both types of observations were pooled. Overall, with 4 TMS pulses per trial, we obtained 60 (15×4) observations per subject and per condition.

### Statistical analysis

A key issue was to assess whether comparable effects of TMS were observed when stimulating M1 and the vertex. To achieve this goal we focused on the comparison between EXT, EXT-M1, and EXT-VERTEX, as well as between SELF, SELF-M1, and SELF-VERTEX. In both cases we used repeated measure ANOVA to assess the effect of experimental conditions. Newman-Keuls corrections were used for post-hoc t-tests to correct for multiple comparisons. A conventional 0.05 significance threshold was used for all analyses.

## Results

**Fig 2:**
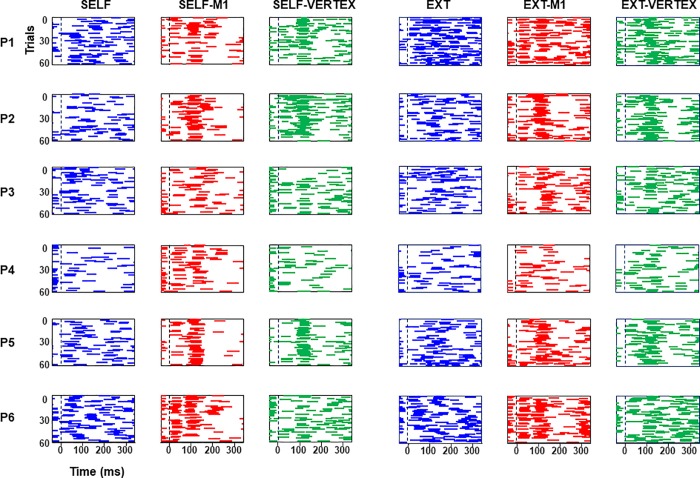
Temporal raster plots of catch-up saccades from all the participants in each experimental condition. Each horizontal segment represents a saccade, and each row represents a trial. All trials are aligned with respect to TMS onset (dotted line), or a comparable moment. Note the drop in saccadic activity followed by a rebound after TMS.

Fig 2 presents raster plots of catch-up saccades for all participants in all the experimental conditions. Although saccades are distributed rather uniformly during SELF and EXTERNAL, one can notice some clear modulations of saccadic activity following TMS over M1 or the VERTEX. Indeed about 40–100 ms after TMS, saccadic activity drops substantially but then strongly resumes within the next 60 ms, and lastly decreases again at about 200–300 ms. To investigate this phenomenon in more detail, we have collapsed all the trials and computed the probability that some saccadic activity is observed within each 20ms time bin following TMS.

### TMS when tracking a self-moved target

**Fig 3:**
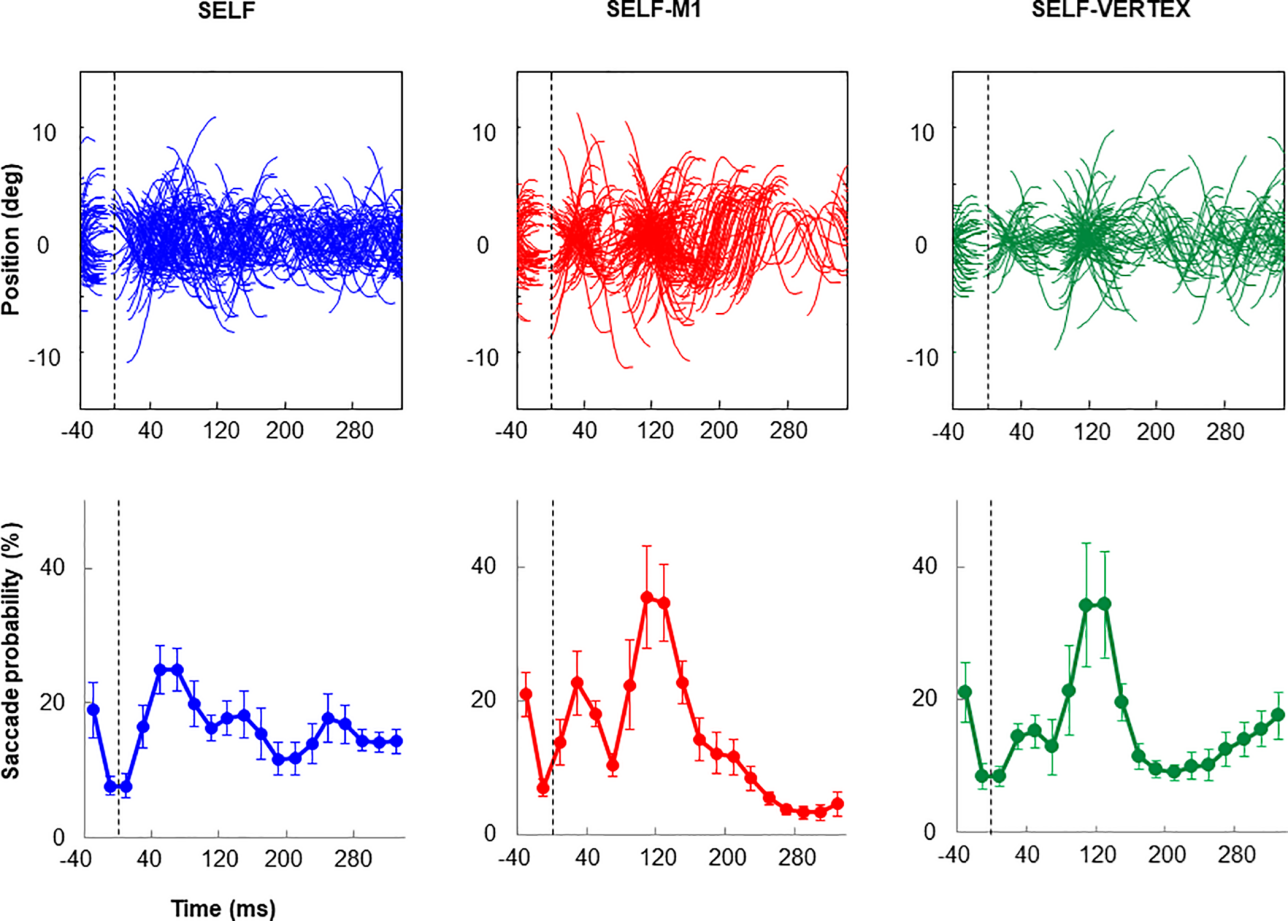
Comparison between the effect of TMS over M1 and the vertex when tracking a self-moved target. A. Saccadic eye signals across all participants and trials (saccades being shifted vertically to be centered around 0°). B. Mean group saccade probability. Data are aligned with respect to TMS onset or a comparable moment (dotted line). In both rows note the clear modulation of saccadic activity following TMS over M1 or the vertex.

The top row of Fig 3 presents all the extracted catch-up saccades obtained from each of the 6 participants (for convenience those saccades were shifted vertically and centered around 0°). Each of the self-moved target condition is displayed on a separate panel. The bottom row presents the corresponding saccade probability within each 20ms time bin. Both rows indicate that, in comparison to SELF (most leftward column), saccadic activity oscillated substantially more in the other two conditions. However, because saccadic activity during SELF was not totally stable, we have decided to subtract this (baseline) activity from the other two conditions so as to circumvent more explicitly the net effect of TMS.

**Fig 4:**
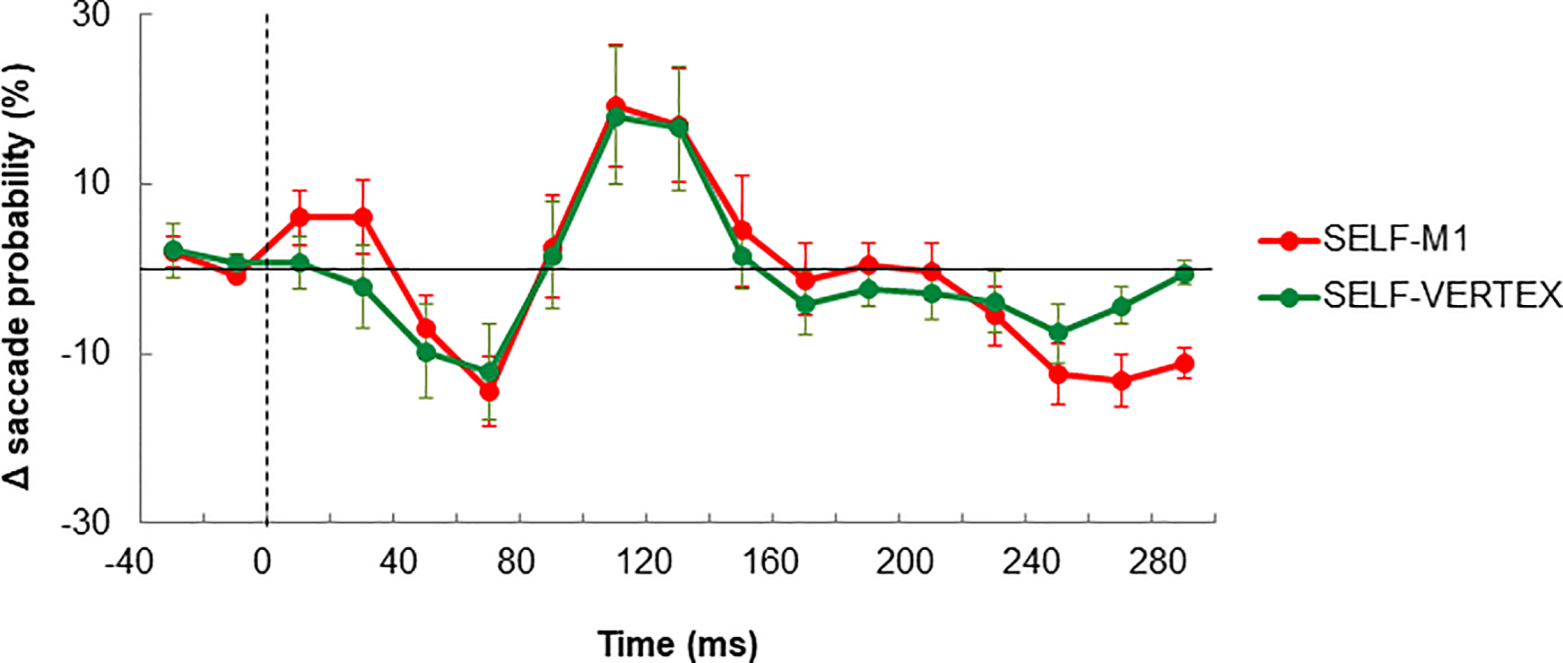
Net effect of TMS over M1 and the vertex on saccade probability when tracking a self-moved target. Data are aligned with respect to TMS onset (dotted line). Note the initial drop in saccadic activity followed by a rebound plus another drop after both types of TMS.

The net effects of TMS on saccade probabilities are presented in Fig 4. Following TMS we clearly see a drop in saccadic activity followed by a rebound, and then another drop. To account for this modulation, using one-way ANOVA, we have compared saccadic activity across our 3 conditions at the specific moments: 70, 130, and 250 ms. In each case results showed an effect of COND (F(2,10)>4.65, p<0.05). Post-hoc analyses indicated that at 70 and 250 ms, saccadic activity was smaller in SELF-M1 and SELF-VERTEX compared to SELF (p<0.05). In contrast, post-hoc analyses revealed that at 130 ms, saccadic activity was greater in SELF-M1 and SELF-VERTEX compared to SELF (p<0.05). For all these three epochs, we found no significant difference between SELF-M1 and SELF-VERTEX (p>0.15) suggesting that this modulatory effect of TMS was similar for M1 and vertex stimulation. Further analyses confirmed that the magnitude of the modulation in saccadic activity, as estimated by the change in saccadic activity between 70 and 130 ms, was similar in SELF-M1 and SELF-VERTEX, reaching respectively 31.5 and 28.6% (F(1,5) = 0.71; p = 0.43).

A subsequent analysis consisted in investigating whether those successive phases of inhibition and facilitation changed the overall saccadic activity during the 0-300ms epoch following TMS. To address this issue we have computed for each condition the mean saccadic probability over that period. One-way ANOVA on this index showed no significant difference across the 3 SELF conditions (F(2,10) = 1.66; p = 0.23). This analysis suggests that, despite some modulations of saccadic activity induced by TMS, the overall amount of saccades initiated during that time window was preserved.

Finally we examined the accuracy of catch-up saccades during SELF and SELF-VERTEX (in which the target trajectory was not perturbed by TMS) at the 3 following epochs: 70, 130 and 250 ms. Two-way ANOVAS (TIME by TMS) showed no significant main effect of TMS (F(1,5) = 1.81; p = 0.24), TIME (F(2,10) = 2.06; p = 0.18), or interaction between the two (F(1,5) = 0.45; p = 0.64). Overall, the mean accuracy of catch-up saccade was 2.1°. Note that we felt it was unfair to include the SELF-M1 condition in this analysis because we know from our previous study [15] that gaze cannot follow the brief target jump induced by TMS, which thereby underestimates the accuracy of catch-up saccade.

### TMS when tracking an externally moved target

Before we address whether the effect of TMS holds when tracking an externally moved target, we would like to stress a key difference in terms of (baseline) saccadic activity. Indeed, although participants initiated on average 3.19 saccades per second in the SELF conditions, this rate increased to 4.17 during the EXTERNAL conditions (+31%; p<0.01). This effect is consistent with other studies [16–18,22,23].

**Fig 5:**
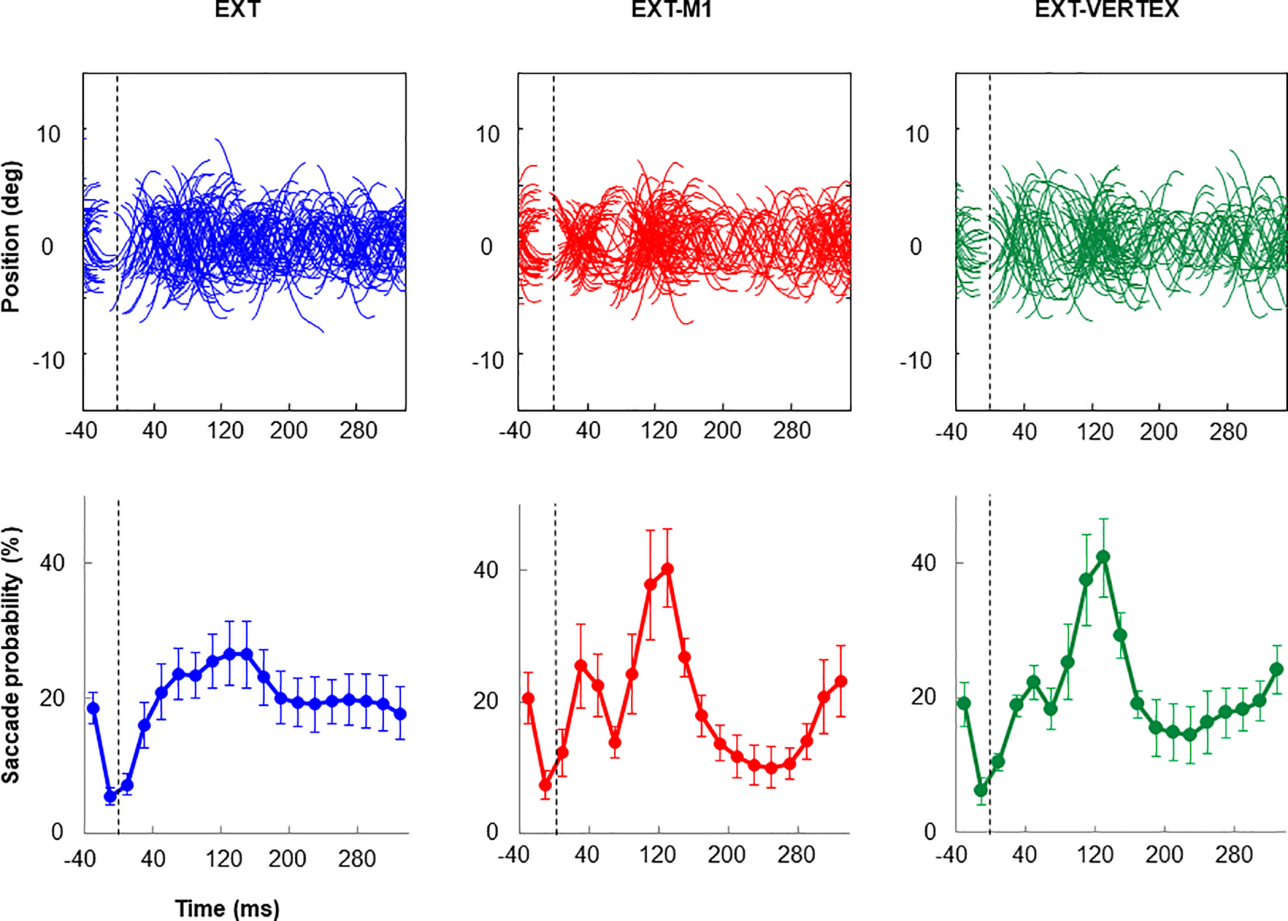
Comparison between the effect of TMS over M1 and the vertex when tracking an externally moved target. A. Saccadic eye signals across all participants and trials (saccades being shifted vertically to be centered around 0°). B. Mean group saccade probability. Data are aligned with respect to TMS onset or a comparable moment (dotted line). Note the modulation of saccadic activity following TMS over M1 or the vertex.

**Fig 6:**
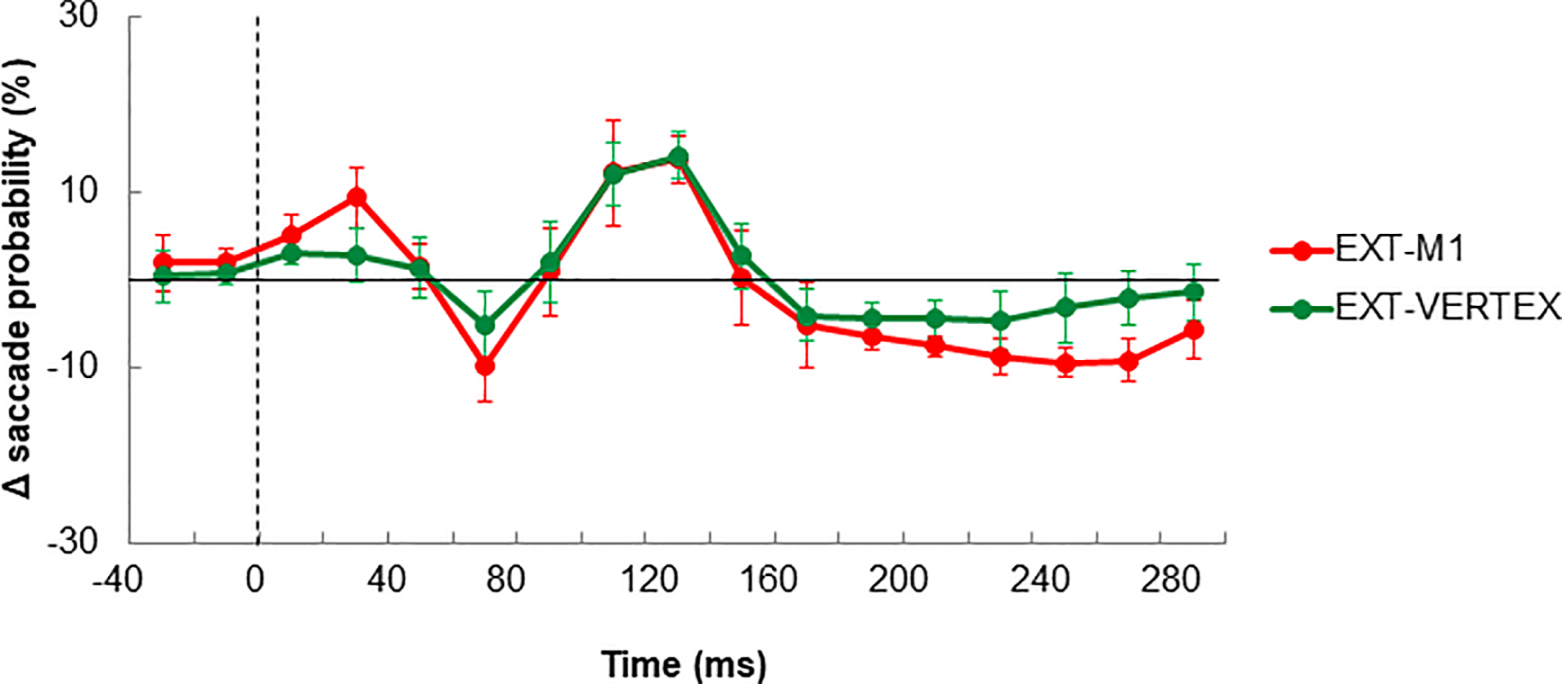
Net effect of TMS over M1 and the vertex on saccade probability when tracking an externally moved target. Data are aligned with respect to TMS onset (dotted line). Note the initial drop in saccadic activity followed by a rebound plus another drop for both types of TMS.

In Fig 5 we present saccadic signals and saccadic probabilities associated with each of the 3 external conditions. As previously reported for the self-moved conditions, visual inspection suggests that TMS was accompanied by a modulation of saccadic activity. To circumvent the net effect of TMS, saccade probabilities observed in EXT were subtracted from those observed in EXT-M1 and EXT-VERTEX. The resulting graphs presented in Fig 6 provide evidence for a similar oscillatory effect of TMS as the one exposed in Fig 4. To account for this modulation, we have run one-way ANOVA comparing our 3 conditions at each of the following epochs: 70, 130, and 230 ms. In each case we found a main effect of COND (F(2,10)>4.34, p<0.05). Post-hoc analyses indicated that at 70 and 230 ms, saccadic activity in EXT-M1 was smaller compared to EXT (p<0.05). A similar tendency was observed when contrasting EXT-VERTEX and EXT, but the effect was marginal (p = 0.06 for 70 ms, and p = 0.11 for 230ms). Examination of the 130 ms epoch revealed greater saccadic activity in EXT-M1 and EXT-VERTEX compared to EXT (p<0.05). For all these three epochs, we found no significant difference between EXT-M1 and EXT-VERTEX (p>0.25) suggesting rather similar effects of TMS for M1 and vertex stimulation. This view is validated by the lack of significant difference between the modulation of saccadic activity in EXT-M1 and EXT-VERTEX (i.e. change in saccadic activity between 70 and 130 ms) reaching respectively 23.4 and 19.5% (F(1,5) = 0.97; p = 0.36). Although seemingly smaller, these variations were comparable to the ones observed during SELF-M1 and SELF-VERTEX (F(3,15) = 1.11; p = 0.37).

As previously done for the SELF conditions, we investigated the overall saccadic activity over the 0–300 ms period following TMS. One-way ANOVA of the mean saccadic probability over that period showed no significant difference across the 3 EXTERNAL conditions (F(2,10) = 0.23; p = 0.79). Finally we compared the accuracy of catch-up saccades in the 3 EXTERNAL conditions at the following epochs: 70, 130 and 230 ms. Again two-way ANOVAS (TIME by TMS) showed no significant main effect of TMS (F(2,10) = 0.41; p = 0.67), TIME (F(2,10) = 0.88; p = 0.44), or interaction (F(4,20) = 1.65; p = 0.20). Overall, the mean accuracy of catch-up saccade was 2.7°.

## Discussion

To our knowledge this study is the first one to explore the effect(s) of TMS on catch-up saccades. At this stage our study brought the following two key observations. First we showed that TMS over M1 and the vertex disrupted the initiation of catch-up saccades no matter whether the target motion was driven by the hand or an external agent. In both cases we observed the same pattern: a depletion in catch-up saccade followed by a rebound, and another depletion. Second, despite those obvious modulations in saccadic activity, we showed that the overall amount of catch-up saccades was preserved, and that their accuracy did not suffer from TMS. We plan now to discuss in more details those findings and their implications.

### TMS alters the initiation of catch-up saccades

We consistently observed that TMS induced a pause in saccadic activity (at 40-100ms) followed by a rebound (at 100-160ms) and a depletion (at 200-300ms). A first possibility to account for these findings is that TMS induced an oscillation in saccadic activity resulting in successive phases of inhibition and facilitation. This possibility is in line with combined TMS/EEG studies suggesting that a single-pulse over M1 could trigger an oscillation and/or reset the ongoing rhythmic activity [24–27]. Furthermore those perturbing effects have been shown to last up to 200 [24] or even 500 ms [25], a time scale that is compatible with our study. Importantly TMS can induce widespread effects on neural oscillations [25,26], and involve after-effects on subcortical structures [25,27], allowing TMS over M1 or the vertex to possibly interfere with oculomotor regions.

Alternatively, rather than successive inhibition/facilitation/inhibition in the generation of catch-up saccades, those modulations in saccadic activity could follow from the fact that TMS is known to interfere with the timing of saccade initiation. As exposed in the introduction, many studies have shown that TMS can effectively delay [3,5,28,29] or shorten [4,6,8,9] the initiation of an upcoming saccade during reaction time protocols. Within this framework, the initial depletion in saccade observed at 70 ms could result from a delay in upcoming saccades, and the rebound seen at 130 ms would emerge from the superposition of those delayed saccades with regular ones. Regarding the second depletion observed at 230–250 ms, this effect is less straightforward but could result from a shortening of saccade latency which thereby also contributed to the preceding rebound. Although this possibility needs to be further explored, this mechanism in which TMS only interfered with the timing of saccade initiation accounts well for the fact that the overall amount of saccades initiated over the 0-300ms period was preserved.

Irrespective of the underlying mechanism, it is also crucial to put forward that TMS induced a pattern of modulation of saccadic activity, whose shape and magnitude, did not vary much across the various contexts tested in this study. Not only the effect was similar for M1 and vertex stimulation, but we also found similar effects no matter whether the target was self or externally driven, despite differences in baseline saccadic activity (30% higher during EXTERNAL). Although these TMS effects have never been described elsewhere, the fact that TMS can interfere with saccadic activity in a similar way from various brain regions has already been reported by Xu-Wilson and colleagues [4]. Indeed, these authors showed similar alterations in saccade latency no matter TMS was applied over the vertex, the cerebellum, the parietal cortex, or the frontal lobe. To account for this observation, they proposed that TMS might produce an activation of the startle system, whose circuitry relies on the reticular formation, a structure that is also involved in the saccadic system [30]. However it is central to point that a loud sound speeds up the initiation of externally guided saccades by about 80 ms [31], an effect consistent with faster releases of hand movements [32,33]. However this effect contrasts markedly with the initial depletion in catch-up saccades observed in our study, making unclear the contribution of a startling effect. Future studies will have to assess this possibility more explicitly.

### TMS does not alter the accuracy of catch-up saccades

It is noteworthy that despite clear modulations in saccadic activity, saccades initiated during the inhibition/facilitation periods landed as close from the target as regular saccades (i.e. initiated without TMS). At first sight this observation seems consistent with the study of Priori and colleagues [3] in which TMS over the vertex delayed saccades but did not alter their accuracy, even though are several differences with respect to our study. First in our study we did not use a fixed target but instead used a moving one, making the accurate landing of saccades more challenging; when considering the mean target velocity in our tasks (about 30°/s), an alteration of 50 ms in the timing of a saccade permits a change of 1.5° in target position. Second, Priori and colleagues used a larger circular coil whose center was placed on the vertex, thereby potentially stimulating FEF and M1. Still, why did not saccade accuracy suffer from TMS? In the context of a neural oscillation, one possibility is that TMS can inhibits/facilitates overall saccadic activity but does not interfere with the actual programming of saccades, allowing them to remain accurate. In the context of TMS altering the timing of saccade initiation, we see two options. A first option is to consider that TMS induces a similar time shift in the preparation and execution of saccades. A second option is to propose that the brain is able to monitor internally this change in timing, like Xu-Wilson and colleagues [4] who observed that TMS-induced perturbation of the eye trajectory was corrected within the same saccade, allowing the eye to reach the (fixed) target. To account for this performance they suggested that, as the saccade unfolds, the brain maintained a real-time estimate of the eye position. However, because our target was constantly moving, the internal monitoring of eye position needs the adjunction of an accurate representation of target trajectory (i.e. to update adequately eye motor commands for the novel target position). Moreover, although the notion of internal model is often evoked for a self-moved target [34–36], this is less obvious for an externally-moved target following a random trajectory [37]. Overall it seems simpler to consider that TMS altered both the programming and the initiation of catch-up saccades. More generally, recent behavioral work suggests that movement preparation and initiation are mechanistically independent and may be subtended by distinct neural bases [38], but here in the context of catch-up saccades, TMS was apparently unable to perturb selectively one of these two operations.

### Differential effects of TMS on smooth pursuit and catch-up saccades

In contrast with our previous study showing no alteration of smooth pursuit with TMS [15], here we show that TMS interferes with catch-up saccades. On the one hand this observation may seem at odd with several evidences of a synergistic behavior between smooth pursuit and saccades [39,40], especially considering that some neural structures, such as the superior colliculus [41] or the oculomotor cerebellar vermis [42,43], are shared by the saccadic and smooth pursuit systems. On the other hand, this observation is reminiscent of another study in which we found an update of smooth pursuit when exposed to a complex hand-target mapping, but no update of catch-up saccades [17]. Altogether these separate behaviors, as evidenced through TMS and an adaptation protocol, question the extent of a synergy between smooth pursuit and catch-up saccade. Our results demonstrate also that, although catch-up saccades are swift movements taking place on the background of a behavior (smooth pursuit) unaffected by TMS, these secondary movements can nevertheless remain sensitive to TMS. One possible reason making saccades more exposed to TMS could stem from the fact that they are driven by a more extended neural network than smooth pursuit [44].

## Conclusion

In this study we showed that TMS over M1 or the vertex were both effective in perturbing the initiation of catch-up saccades, even though these two regions are rarely evoked for eye movements. As a result this study puts forward TMS as novel tool for perturbing catch-up saccades. Whether TMS over more traditional oculomotor regions (FEF or cerebellum) would be as effective, or possibly more effective, is still unknown and should be addressed in future studies. Finally, despite several limitations making unclear the exact neural mechanism underlying the effect of TMS on catch-up saccades, our study emphasizes that this effect is robust (i.e. task independent) and therefore should be considered by future studies that wish to investigate the neural circuitry of catch-up saccades with TMS. In that sense, we hope this study provides tutorial guidance and methodological considerations for upcoming studies.

## References

[1] Colnaghi S, Ramat S, D’Angelo E, Versino M. Transcranial magnetic stimulation over the cerebellum and eye movements: state of the art. Funct Neurol. 2010;25: 165–171. 21232213

[2] Vernet M, Quentin R, Chanes L, Mitsumasu A, Valero-Cabré A. Frontal eye field, where art thou? Anatomy, function, and non-invasive manipulation of frontal regions involved in eye movements and associated cognitive operations. Front Integr Neurosci. 2014;8: 66 10.3389/fnint.2014.00066 25202241 PMC4141567

[3] Priori A, Bertolasi L, Rothwell JC, Day BL, Marsden CD. Some saccadic eye movements can be delayed by transcranial magnetic stimulation of the cerebral cortex in man. Brain J Neurol. 1993;116 (Pt 2): 355–367. 10.1093/brain/116.2.355 8461970

[4] Xu-Wilson M, Tian J, Shadmehr R, Zee DS. TMS perturbs saccade trajectories and unmasks an internal feedback controller for saccades. J Neurosci. 2011;31: 11537–11546. 10.1523/JNEUROSCI.1584-11.2011 21832184 PMC3167087

[5] Thickbroom GW, Stell R, Mastaglia FL. Transcranial magnetic stimulation of the human frontal eye field. J Neurol Sci. 1996;144: 114–118. 8994112 10.1016/s0022-510x(96)00194-3

[6] Nyffeler T, Bucher O, Pflugshaupt T, Von Wartburg R, Wurtz P, Hess CW, et al Single-pulse transcranial magnetic stimulation over the frontal eye field can facilitate and inhibit saccade triggering. Eur J Neurosci. 2004;20: 2240–2244. 10.1111/j.1460-9568.2004.03667.x 15450104

[7] Valero-Cabre A, Wattiez N, Monfort M, François C, Rivaud-Péchoux S, Gaymard B, et al Frontal non-invasive neurostimulation modulates antisaccade preparation in non-human primates. PloS One. 2012;7: e38674 10.1371/journal.pone.0038674 22701691 PMC3368878

[8] Zangemeister WH, Nagel M. Transcranial magnetic stimulation over the cerebellum delays predictive head movements in the coordination of gaze. Acta Oto-Laryngol Suppl. 2001;545: 140–144. 10.1080/000164801750388324 11677729

[9] Nagel M, Zangemeister WH. The effect of transcranial magnetic stimulation over the cerebellum on the synkinesis of coordinated eye and head movements. J Neurol Sci. 2003;213: 35–45. 12873753 10.1016/s0022-510x(03)00145-x

[10] Gagnon D, Paus T, Grosbras M-H, Pike GB, O’Driscoll GA. Transcranial magnetic stimulation of frontal oculomotor regions during smooth pursuit. J Neurosci. 2006;26: 458–466. 10.1523/JNEUROSCI.2789-05.2006 16407543 PMC6674407

[11] Nuding U, Kalla R, Muggleton NG, Büttner U, Walsh V, Glasauer S. TMS evidence for smooth pursuit gain control by the frontal eye fields. Cereb Cortex. 2009;19: 1144–1150. 10.1093/cercor/bhn162 18832331

[12] Ohtsuka K, Enoki T. Transcranial magnetic stimulation over the posterior cerebellum during smooth pursuit eye movements in man. Brain. 1998;121 (Pt 3): 429–435. 9549519 10.1093/brain/121.3.429

[13] Haarmeier T, Kammer T. Effect of TMS on oculomotor behavior but not perceptual stability during smooth pursuit eye movements. Cereb Cortex. 2010;20: 2234–2243. 10.1093/cercor/bhp285 20064941

[14] de Brouwer S, Yuksel D, Blohm G, Missal M, Lefèvre P. What triggers catch-up saccades during visual tracking? J Neurophysiol. 2002;87: 1646–1650. 10.1152/jn.00432.2001 11877535

[15] Mathew J, Eusebio A, Danion F. Limited Contribution of Primary Motor Cortex in Eye-Hand Coordination: A TMS Study. J Neurosci. 2017;37: 9730–9740. 10.1523/JNEUROSCI.0564-17.2017 28893926 PMC6596613

[16] Chen J, Valsecchi M, Gegenfurtner KR. LRP predicts smooth pursuit eye movement onset during the ocular tracking of self-generated movements. J Neurophysiol. 2016;116: 18–29. 10.1152/jn.00184.2016 27009159 PMC4961754

[17] Landelle C, Montagnini A, Madelain L, Danion F. Eye tracking a self-moved target with complex hand-target dynamics. J Neurophysiol. 2016;116: 1859–1870. 10.1152/jn.00007.2016 27466129 PMC5144706

[18] Steinbach MJ, Held R. Eye tracking of observer-generated target movements. Science. 1968;161: 187–188. 5657071 10.1126/science.161.3837.187

[19] Rossi S, Hallett M, Rossini PM, Pascual-Leone A, Safety of TMS Consensus Group. Safety, ethical considerations, and application guidelines for the use of transcranial magnetic stimulation in clinical practice and research. Clin Neurophysiol. 2009;120: 2008–2039. 10.1016/j.clinph.2009.08.016 19833552 PMC3260536

[20] Brasil-Neto JP, Cohen LG, Panizza M, Nilsson J, Roth BJ, Hallett M. Optimal focal transcranial magnetic activation of the human motor cortex: effects of coil orientation, shape of the induced current pulse, and stimulus intensity. J Clin Neurophysiol. 1992;9: 132–136. 1552001

[21] Mills KR, Boniface SJ, Schubert M. Magnetic brain stimulation with a double coil: the importance of coil orientation. Electroencephalogr Clin Neurophysiol. 1992;85: 17–21. 1371739 10.1016/0168-5597(92)90096-t

[22] Angel RW, Garland H. Transfer of information from manual to oculomotor control system. J Exp Psychol. 1972;96: 92–96. 5083135 10.1037/h0033457

[23] Mather J, Lackner J. Adaptation to visual rearrangement elicited by tonic vibration reflexes. Exp Brain Res. 1975;24: 103–105. 128465 10.1007/BF00236021

[24] Paus T, Sipila PK, Strafella AP. Synchronization of neuronal activity in the human primary motor cortex by transcranial magnetic stimulation: an EEG study. J Neurophysiol. 2001;86: 1983–1990. 10.1152/jn.2001.86.4.1983 11600655

[25] Fuggetta G, Fiaschi A, Manganotti P. Modulation of cortical oscillatory activities induced by varying single-pulse transcranial magnetic stimulation intensity over the left primary motor area: a combined EEG and TMS study. NeuroImage. 2005;27: 896–908. 10.1016/j.neuroimage.2005.05.013 16054397

[26] Van Der Werf YD, Paus T. The neural response to transcranial magnetic stimulation of the human motor cortex. I. Intracortical and cortico-cortical contributions. Exp Brain Res. 2006;175: 231–245. 10.1007/s00221-006-0551-2 16783559

[27] Van Der Werf YD, Sadikot AF, Strafella AP, Paus T. The neural response to transcranial magnetic stimulation of the human motor cortex. II. Thalamocortical contributions. Exp Brain Res. 2006;175: 246–255. 10.1007/s00221-006-0548-x 16832683

[28] Ro T, Henik A, Machado L, Rafal RD. Transcranial magnetic stimulation of the prefrontal cortex delays contralateral endogenous saccades. J Cogn Neurosci. 1997;9: 433–440. 10.1162/jocn.1997.9.4.433 23968209

[29] Nagel M, Sprenger A, Lencer R, Kömpf D, Siebner H, Heide W. Distributed representations of the “preparatory set” in the frontal oculomotor system: a TMS study. BMC Neurosci. 2008;9: 89 10.1186/1471-2202-9-89 18801205 PMC2564971

[30] Büttner-Ennever J, Horn A. Reticular formation: eye movements, gaze, and blinks The human nervous system. (Paxinos G, Mi Jk, eds). Amsterdam: Elsevier; 2004 pp. 479–510.

[31] Castellote JM, Kumru H, Queralt A, Valls-Solé J. A startle speeds up the execution of externally guided saccades. Exp Brain Res. 2007;177: 129–136. 10.1007/s00221-006-0659-4 16944110

[32] Valls-Solé J, Rothwell JC, Goulart F, Cossu G, Muñoz E. Patterned ballistic movements triggered by a startle in healthy humans. J Physiol. 1999;516 (Pt 3): 931–938. 10200438 10.1111/j.1469-7793.1999.0931u.x PMC2269293

[33] Carlsen AN, Chua R, Inglis JT, Sanderson DJ, Franks IM. Prepared movements are elicited early by startle. J Mot Behav. 2004;36: 253–264. 10.3200/JMBR.36.3.253-264 15262622

[34] Scarchilli K, Vercher JL, Gauthier GM, Cole J. Does the oculo-manual co-ordination control system use an internal model of the arm dynamics? Neurosci Lett. 1999;265: 139–142. 10327188 10.1016/s0304-3940(99)00224-4

[35] Ariff G, Donchin O, Nanayakkara T, Shadmehr R. A real-time state predictor in motor control: study of saccadic eye movements during unseen reaching movements. J Neurosci. 2002;22: 7721–7729. 12196595 10.1523/JNEUROSCI.22-17-07721.2002 PMC6757993

[36] Vercher J-L, Sarès F, Blouin J, Bourdin C, Gauthier G. Role of sensory information in updating internal models of the effector during arm tracking. Prog Brain Res. 2003;142: 203–222. 10.1016/S0079-6123(03)42015-3 12693263

[37] Goffart L, Bourrelly C, Quinet J. Synchronizing the tracking eye movements with the motion of a visual target: Basic neural processes. Prog Brain Res. 2017;236: 243–268. 10.1016/bs.pbr.2017.07.009 29157414

[38] Haith AM, Pakpoor J, Krakauer JW. Independence of Movement Preparation and Movement Initiation. J Neurosci. 2016;36: 3007–3015. 10.1523/JNEUROSCI.3245-15.2016 26961954 PMC6601759

[39] Orban de Xivry J-J, Bennett SJ, Lefèvre P, Barnes GR. Evidence for synergy between saccades and smooth pursuit during transient target disappearance. J Neurophysiol. 2006;95: 418–427. 10.1152/jn.00596.2005 16162830

[40] Orban de Xivry J-J, Lefèvre P. Saccades and pursuit: two outcomes of a single sensorimotor process. J Physiol. 2007;584: 11–23. 10.1113/jphysiol.2007.139881 17690138 PMC2277072

[41] Krauzlis RJ, Basso MA, Wurtz RH. Discharge properties of neurons in the rostral superior colliculus of the monkey during smooth-pursuit eye movements. J Neurophysiol. 2000;84: 876–891. 10.1152/jn.2000.84.2.876 10938314

[42] Takagi M, Zee DS, Tamargo RJ. Effects of lesions of the oculomotor vermis on eye movements in primate: saccades. J Neurophysiol. 1998;80: 1911–1931. 10.1152/jn.1998.80.4.1911 9772249

[43] Takagi M, Zee DS, Tamargo RJ. Effects of lesions of the oculomotor cerebellar vermis on eye movements in primate: smooth pursuit. J Neurophysiol. 2000;83: 2047–2062. 10.1152/jn.2000.83.4.2047 10758115

[44] Krauzlis RJ. Recasting the smooth pursuit eye movement system. J Neurophysiol. 2004;91: 591–603. 10.1152/jn.00801.2003 14762145

